# Spontaneous haemorrhagic cholecystitis secondary to the use of novel anticoagulants (rivaroxaban)

**DOI:** 10.1259/bjrcr.20220128

**Published:** 2023-01-11

**Authors:** Merina Kurian, Chee Khong Lim, Prabhjoyt Kler, Bing Lun Chow, Cyril Jacob Chacko

**Affiliations:** 1 Royal Wolverhampton NHS Trust, London, UK; 2 Russells Hall Hospital NHS, London, UK

## Abstract

Haemorrhagic cholecystitis is a rare complication of acute cholecystitis. It carries a high risk of morbidity and mortality. Risk factors for haemorrhagic cholecystitis include cholelithiasis, trauma, malignancy and the use of anticoagulants.

There have only been a few reported cases of haemorrhagic cholecystitis secondary to the use of novel oral anticoagulants (NOACs). The demographic transition of an ageing population will potentially increase the utilisation of NOACs. Therefore, the incidence of haemorrhagic cholecystitis secondary to NOACs will likely increase. Awareness and prompt diagnosis is paramount to avoid morbidity and mortality associated with haemorrhagic cholecystitis.

## Clinical presentation

A 74-year-old gentleman presented with a history of vomiting, pyrexia and intermittent epigastric pain which began days prior to his admission.His previous medical history included ischaemic stroke, atrial fibrillation and recent COVID pneumonitis.He was on rivaroxaban for atrial fibrillation.

## Investigations/Imaging findings

The patient was hypotensive on presentation. Salient examination findings at presentation included generalised abdominal distension and tenderness with a positive Murphy’s sign. Blood test results were as follows:

1. Acute cholecystitis
2. Acute pancreatitis
3. Acute cholangitis
4. Choledocholithiasis
5. Pancreatic or ampullar malignancy

White blood cell count (WBCC): 16.3

C-reactive protein (CRP): 252

Bilirubin: 40

Erythrocyte sedimentation rate (ESR): 60

Haemoglobin (Hb): 99 gl (previously 111)

The blood test results of high WBCC, ESR and CRP were suggestive of an acute underlying infective or inflammatory process. The raised bilirubin was suggestiveof involvement of the biliary system. The drop in Hb from 111 to 99 indicated acute blood loss.

The patient had a clinical history of gallstones diagnosed on a previous ultrasound scan. As such, the following differential diagnoses were considered likely on presentation:

1. Acute cholecystitis
2. Acute pancreatitis
3. Acute cholangitis
4. Choledocholithiasis
5. Pancreatic or ampullar malignancy

**Figure 1.**
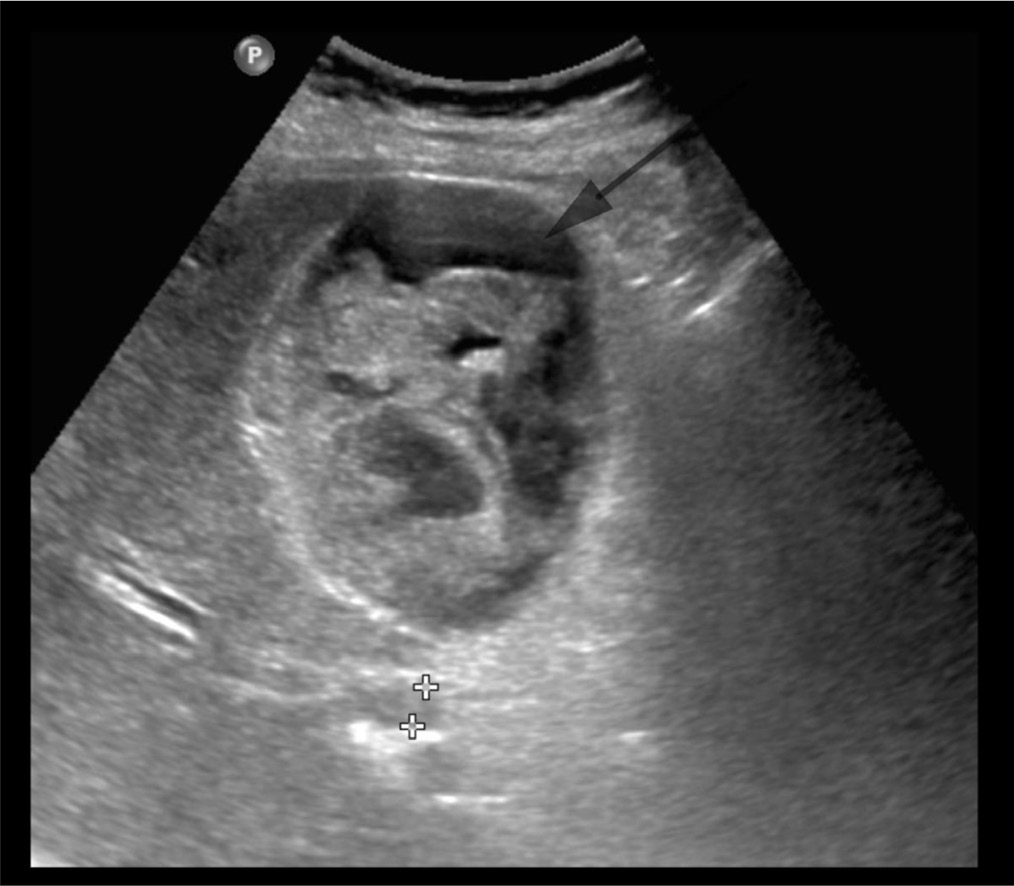
Ultrasound abdomen and pelvis

An abdominal ultrasound scan was performed (Figure 1). This scan showed gallstones, distended thick-walled gallbladder, and intraluminal hyperechoic changes, raising suspicion for empyema of the gallbladder.

**Figure 2.**
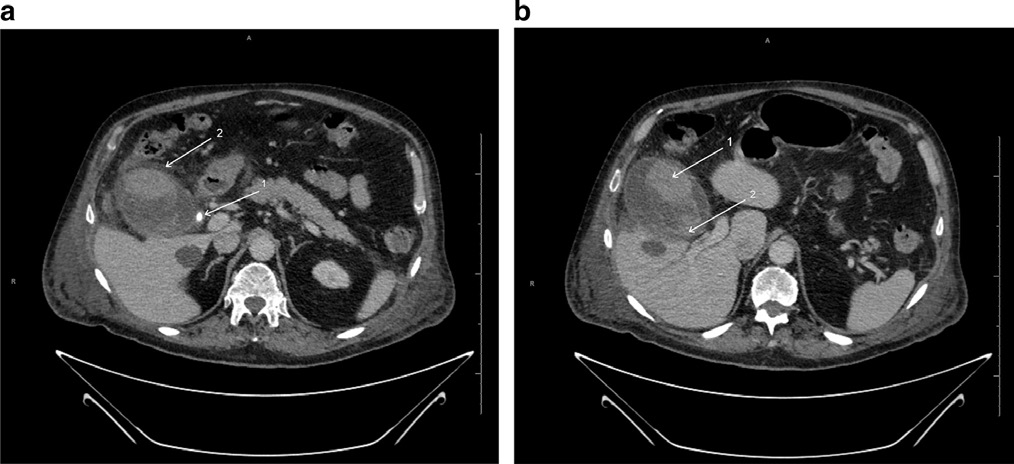
a.Contrast enhanced CT of the abdomen and pelvis. 2b. Contrast enhanced CT of the abdomen and pelvis

Subsequently, the patient had a contrast-enhanced CT of the abdomen and pelvis Figure 2. This showed features of calculous cholecystitis, with gallstones and inflamed gallbladder. Intraluminal high attenuation changes seen in addition to gallstones were considered to be changes related to acute cholecystitis. The patient was managed conservatively due to associated co-morbidities.

**Figure 3.**
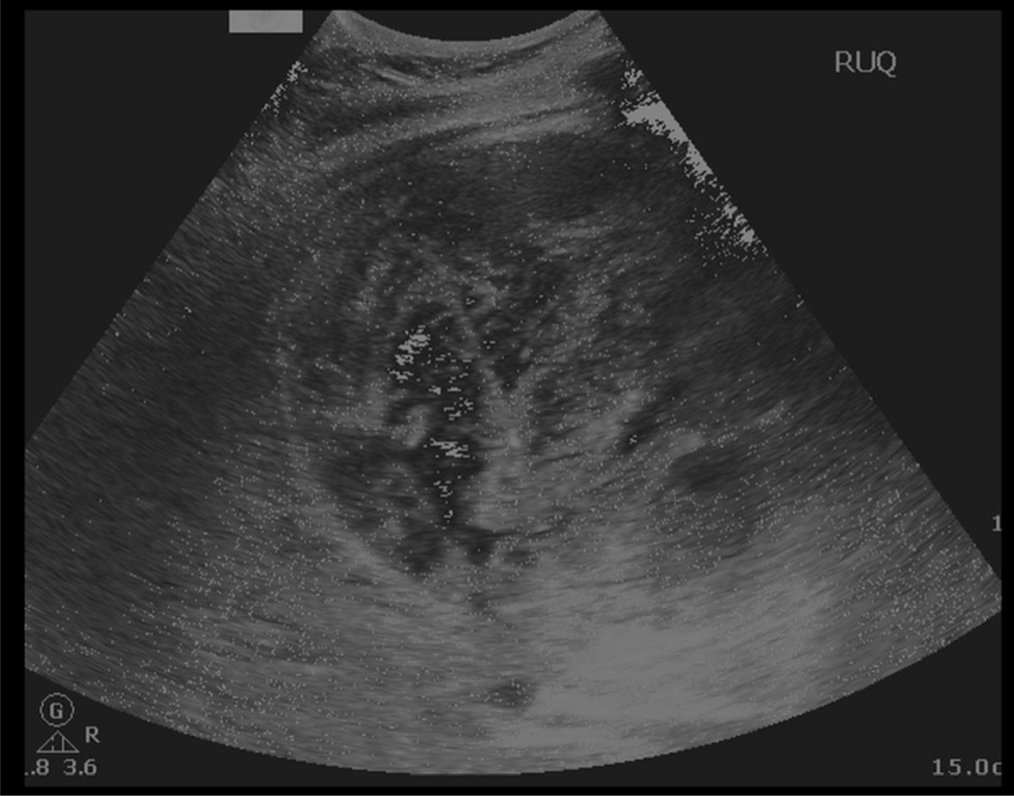
Ultrasound abdomen and pelvis

The patient was booked for an ultrasound-guided cholecystostomy (Figure 3) as he continued to deteriorate with persistently raised inflammatory markers. The ultrasound showed a large distended gallbladder with intraluminal hyperechoic changes. The cholecystostomy aspirated blood.

**Figure 4.**
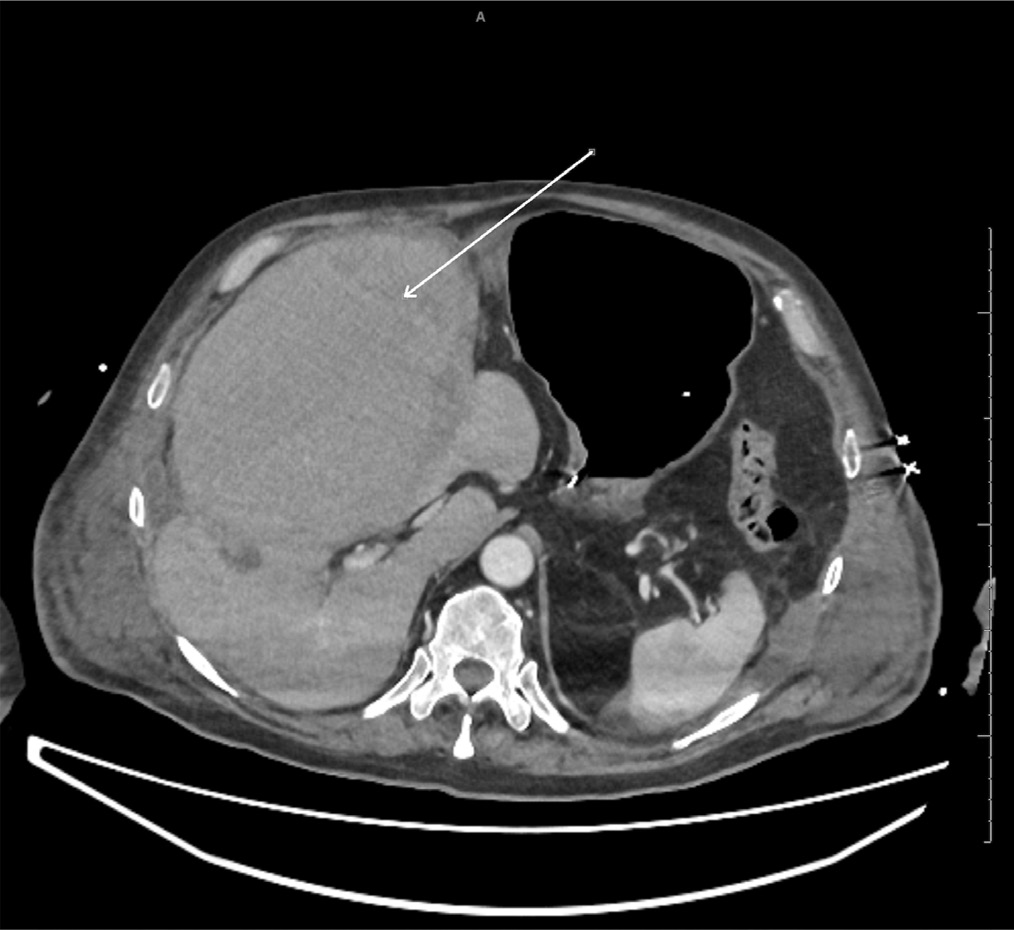
CT angiogram

Three days after the cholecystostomy, the patient had a CT angiogram performed due to the drop in Hb (Figure 4). This showed a massively distended gallbladder with intraluminal high attenuation changes.This was followed by an ERCP due to the raised bilirubin. The ERCP showed blood at the duodenal ampulla.

## Treatment

A diagnosis of haemorrhagic cholecystitis secondary to NOACuse was made based on the imaging findings. The patient’s anticoagulation was reversed. Ultrasound-guided percutaneous cholecystostomy drained blood. The patient’s symptoms improved and he was subsequently discharged home without further complications. Follow-up ultrasound scan after 2 months showed improvement in the appearance of the gallbladder with mild residual changes.

## Discussion

A literature review was conducted. Search engines such as PubMed and Medline were utilised. Search terms included NOAC, haemorrhagic cholecystitis and management. Cases from 1990 onwards were included.

Haemorrhagic cholecystitis is a rare complication of acute cholecystitis thought to arise from vessel destruction and mucosal infarction secondary to chronic inflammation of the gallbladder. The usage of anticoagulants is a known risk factor for haemorrhagic cholecystitis.^1^ Other reported causes for haemorrhagic cholecystitis include iatrogenic causes, bleeding diathesis, inflammation, trauma, renal failure and even malignancy.^2^ Prompt diagnosis is crucial given the morbidity and mortality which is reported to be 15–20%.^3^

The diagnosis of haemorrhagic cholecystitis can be challenging as it is a great mimic of other right upper quadrant pathology. The distension of gallbladder and ongoing inflammation may mimic acute cholecystitis. In severe cases, blood may enter the gastrointestinal tract and manifest as melena as well as haematemesis. This may also be further complicated by haemoperitoneum secondary to gallbladder rupture.

The classical presentation of jaundice, right upper quadrant pain and upper gastrointestinal bleeding, known as Quincke’s triad only occurs in approximately 22% of patients.^2,4^ Even laboratory findings could be misleading with leucocytosis and abnormal liver function tests.

Radiological investigations play a crucial role in the acute setting and can aid prompt diagnosis.Imaging modalities such as ultrasonography, CT, MRI and radionuclide imaging have been described in literature to aid the diagnosis of haemorrhagic cholecystitis.

Sonographic findings of haemorrhagic cholecystitis include thickened gallbladder wall, intraluminal hyperechogenicity and non-shadowing coarse echoes.^5^ Intraluminal hyperechoic changes with supporting clinical history should raise the suspicion of haemorrhagic cholecystitis.

CT imaging is usually carried out following an ultrasound scan to confirm the diagnosis of acute cholecystitis and assess for any associated complications. The presence of irregular intraluminal high attenuation changes with layering in conjunction with typical findings of acute cholecystitis, should raise the suspicion for haemorrhagic cholecystitis.

MRI imaging may not be the appropriate for first-line investigation as arranging the scan may result in delayed intervention. Findings on MRI include endoluminal T1 fat-suppressed hyperintensity with variable T2 signal intensity.^4^

In summary, there should be a high index of suspicion for haemorrhagic cholecystitis in patients with acute cholecystitis on NOACs, with a drop in Hb. Clinical and radiological awareness of this rare but fatal complication will help with prompt management of this condition.

## Learning points

- Haemorrhagic cholecystitis is a rare but life-threatening complication of acute cholecystitis of which NOAC use is a risk factor.
- Clinical diagnosis can be challenging, however a drop in Hb in appropriate patients should raise the suspicion of haemorrhagic cholecystitis.
- Diagnosis can only be made on imaging investigations.
- Awareness of this rare but fatal complication among clinicians and radiologists should lead to early investigations and accurate interpretation of the imaging findings.
